# Virtual Reality and Relaxation for the Treatment of Generalized Anxiety Disorder: A Randomized Comparative Study with Standard Intervention

**DOI:** 10.3390/jcm14041351

**Published:** 2025-02-18

**Authors:** Eric Malbos, Nadège Chichery, Baptiste Borwell, Gabriel Weindel, Jordan Molitor, Mélodie Einig-Iscain, Julien Seimandi, Christophe Lançon

**Affiliations:** 1 Department of Adult Psychiatry, Sainte Marguerite University Hospital, Assistance Publique Hôpitaux de Marseille APHM, 13009 Marseille, France; 2 Equipe Imothep, Institut Fresnel, UMR 7249, Ecole Centrale Marseille, Aix-Marseille University, 13005 Marseille, France; 3 Laboratory of Cognitive Neuroscience, UMR 7291, Aix-Marseille University, 13331 Marseille, France; 4 Centre de Recherche en Santé Mentale et Psychiatrie, 13090 Montperrin, France

**Keywords:** virtual reality, relaxation therapy, generalized anxiety disorder, digital health, virtual environments, presence

## Abstract

**Background/Objectives:** Modern therapeutic strategies incorporating virtual reality (VR) have emerged as potential treatments for generalized anxiety disorder (GAD), a prevalent and debilitating condition that is challenging to cure. This study aimed to evaluate the efficacy of VR combined with relaxation techniques in patients with GAD by comparing VR-based relaxation with standard mental imagery (MI) relaxation. **Methods:** Fifty-eight patients with GAD participated in a randomized comparative trial. Specific virtual environments were created using an inexpensive game engine/level editor (GLE). Psychometric scales and physiological instruments were employed to assess the effects of relaxation therapy on anxiety, depression, quality of life, presence within virtual environments and cybersickness. **Results:** Both the VR and MI groups demonstrated statistically significant improvements in anxiety, worry and mental quality of life scores. However, no significant differences were observed between the two groups in pre–post comparisons of psychometric scores. The VR group exhibited a noticeably higher protocol completion rate and a significant increase in heart rate variability during the therapy. The level of presence in the VR group was satisfactory and significantly correlated with physiological improvements and anxiety reduction, while cybersickness remained low. Participants’ preferences for specific virtual environments for relaxation are also discussed. **Conclusions:** These findings suggest that teaching and practicing relaxation in VR holds therapeutic potential for the treatment of GAD. Further research leveraging advanced VR sensory equipment and artificial intelligence agents is warranted to enhance therapeutic outcomes and explore additional applications.

## 1. Introduction

The increasing use of and access to virtual reality (VR) technologies and digital health for clinicians as well as for the general population and its use in mental health structures or at home in various contexts (i.e., games, education) have led to the production of meditation or relaxation VR software and serious games dedicated to stress or anxiety management. While anxiety is a physiological phenomenon allowing an individual to cope with a threatening situation, when anxiety is excessive or prolonged, it is then regarded as pathological [1]. The World Health Organization (WHO) reported that, in 2015, anxiety disorders ranked in sixth place among all mental and somatic illnesses worldwide as a cause of so-called years lived with disability (YLD), and in fourth place in highly developed countries; they are therefore among the chronic illnesses with the greatest impact on patients’ lives [2]. What the media and general population refer to as “anxiety” is often an anxiety disorder associated with chronic worry, entitled generalized anxiety disorder (GAD). It is a frequent and disabling condition which affects approximately 6.2% of the general population in France, 7.8% in the USA and 8% in Australia [3] (lifetime prevalence for all). It is described as excessive anxiety and worry (apprehensive expectation) occurring more days than not, for at least six months, about a number of events or activities (such as work or school performance) [1]. Aside from anxiety, various symptoms can be noted such as muscle tension and cardiac or digestive discomfort [4]. GAD has a long-term evolution and a major impact on functional capacity for patients, especially when considering the frequent comorbidity with other psychiatric illnesses (anxiety and mood disorders) [5]. It affects quality of life, with severe consequences on health and financial issues [6].

Various therapeutic options have been assessed for the treatment of this disorder. Cognitive behavior therapy (CBT) and relaxation methods are recommended as first-line treatments [7]. Indeed, relaxation, compared to CBT, has proven its efficacy in the short-term treatment of GAD [8]. Different relaxation techniques are available to therapists: respiratory control methods [9], with either abdominal breathing or equal breathing techniques; progressive muscular relaxation [10]; applied relaxation [11]; autogenic training [12]; and meditation, in particular mindfulness [13].

Although there is strong evidence that CBT and relaxation techniques have beneficial effects on GAD symptoms [14], some patients still experience considerable disability and impaired quality of life, as monotherapy may be insufficient to manage the whole spectrum of this mental disorder [15]. Consequently, other tools derived from technological improvements and CBT techniques have been developed for the treatment of GAD, and most noticeably, procedures involving virtual reality. When applied to other anxiety disorders, this method is known as Virtual Reality Exposure Therapy (VRET). VRET exposes the patient to 3D computer-generated virtual environments. It has already shown promising results in the treatment of anxiety related disorders such as specific phobias (aerophobia, aviophobia, arachnophobia), social phobia, panic disorder, agoraphobia and post-traumatic stress disorder [16,17]. Similarly, patients suffering from GAD can be exposed to relaxing, soothing or calming environments created by computers in the context of Virtual Reality Relaxation Therapy (VRT).

However, studies on relaxation in virtual reality are but a few and remain inconclusive to support the superiority of VR as a substitute for therapeutic approaches [18], while some have questioned the reliability of the registered effects [19]. Research teams have devised protocols to assess the potential of virtual reality as a stress management therapy with progressive relaxation in GAD [20]. For instance, Gorini et al. developed a protocol employing a virtual environment simulating a tropical island to guide users through predefined circuits and relaxation exercises with biofeedback integration [21]. Other investigations have explored distinct approaches, such as employing catastrophic VR scenarios [22] or VR-based worry exposure [23]. With regard to platforms, Grassi et al. used different virtual environments (the “Green Valley”) in order to test stress reduction through mobile devices such as tablets and smartphones [24]. Recognizing the potential impact of physical activity on mental health, Wang et al. conducted a study incorporating cycling within virtual environments, yielding promising results in mitigating emotional stress [25]. A more integrative study combined VR and CBT, autogenic training relaxation and meditation for treating GAD, with positive results on symptoms and worries [26]. However, to our knowledge, no controlled study with group comparisons has specifically investigated the effects of VR therapy (VRT) on anxiety using three established relaxation techniques (abdominal vagal breathing, progressive muscular relaxation and autogenic training) while offering participants a selection of specially designed interactive virtual environments and an analysis of the level of immersion or implication associated with this method. It is noteworthy to stress the term “interactive” as contrary to numerous relaxing 360° video recordings that can be found on the internet wherein the user is a passive observer of pre-recorded landscapes; interactive virtual environments empower users to actively engage by navigating the digital world: walking, climbing, swimming, flying or interacting with 3D objects in real time. This active participation fosters a sense of agency, providing users with greater autonomy and control over their virtual experiences.

Therefore, the main objective of this study was to further previous works by assessing and comparing the therapeutic efficacy of VRT and the use of six interactive and calming virtual environments created with a GLE as a means of learning and practicing three different established relaxation techniques, without biofeedback, for patients suffering from GAD. Thus, the potential effects were measured with the PSWQ (Penn State Worry Questionnaire) score as the primary outcome, along with other related psychometric scales and physiological recordings linked to anxiety and analyzed between the first and last sessions of the present protocol. Participants were randomly assigned to one of two groups to compare the outcomes of VRT with those of traditional relaxation techniques involving mental imagery (MI). The secondary objective was to evaluate and compare the impact of the VR-based therapeutic strategy on additional psychological dimensions, including mood and quality of life. The third objective was to measure the intensity of presence exhibited among the participants of the VRET group when they were immersed in the supposedly relaxing virtual environments specifically constructed for this study. A key to successful immersion and therapy is this sense of presence, which has been frequently defined as a transportation medium: a sensation of ‘being there’ within a virtual world [27].

## 2. Materials and Methods

The present trial was registered on ClinicalTrials.gov (Identifier: NCT02571790).

### 2.1. Sample

The required sample size of 58 subjects was determined based on power calculations for a repeated-measures ANOVA assessing both the main effect of time and the time × group interaction, with the primary outcome being the Penn State Worry Questionnaire (PSWQ) score. Assuming a moderate effect size (Cohen’s f = 0.25, corresponding approximately to a partial η^2^ of 0.06), a 10% risk of dropout, an alpha level of 0.05 and a desired power of 0.95, power analyses were conducted using G*Power (version 3.0).

The participants (33 female, 25 male) meeting the DSM-5 criteria for GAD were recruited for the clinical trial through local media and onsite consultations. The inclusion criteria were as follows: male or female, aged between 18 and 70 years old, with a primary diagnosis of GAD as defined by the DSM-5 [1]. The exclusion criteria included pregnancy, comorbid severe clinical depression (BDI score ≥ 29), concurrent CBT, medication changes in the month previous to inclusion, history of active neurological disease, severe head trauma, intellectual disability or any major untreated physical disorder, any untreated psychotic disorder, addiction or any contraindications to virtual reality (migraine, photosensitive epilepsy, vestibular disorder).

Diagnoses were established by the first author based on a semi-structured interview, the Anxiety Disorders Interview Schedule for DSM-5 (ADIS-5) [7]. Sociodemographic characteristics are listed in Table 1.

### 2.2. Assessments

Pre- and post-treatment measures were assessed using self-rated anxiety, worry, depression and quality of life questionnaires with the following self-report scales:

State Trait Anxiety Inventory (STAI Y-A and Y-B) [28]. This includes forty items seeking to evaluate the degree of intensity or the frequency of the patient’s anxious state using a four-point scale. The score ranges from 20 (very low anxiety) to 80 (very high anxiety). Form A specifically assesses state-anxiety at the time of the test, and form B assesses trait-anxiety in general.

Beck’s Depression Inventory version II (BDI-II) [29]. This scale allows for a quick self-evaluation of depression symptoms. Each item consists of four sentences of increasing symptom intensity, graded between 0 and 3. The overall score is calculated by adding together the individual scores of the 13 items.

Penn State Worry Questionnaire (PSWQ) [30]. This questionnaire assesses the level of worry of patients suffering from GAD. It includes sixteen items seeking to assess the level of worry of patients suffering from GAD. Participants had to grade each item with a mark ranging from 1 (no worry) to 5 (extreme worry). This scale offered good reliability and validity, which enabled us to differentiate between patients suffering from GAD and patients suffering from other anxiety disorders. The maximum score, which corresponds to the total added scores of the patient, is 80 (very high worry).

SF-12 quality of life questionnaire [31]. Quality of life was assessed with this 12-item scale assessing physical function, physical pain, general health, vitality, social functioning and well-being. Each answer is weighted for every study patient so as to obtain a Physical Component Score (PCS) and a Mental Component Score (MCS).

Aside from pre–post measures, the Subjective Units of Discomfort (SUDs) [32] were recorded at three regular intervals throughout the VR sessions. This is a 10 or 100 point scale test which measures the perceived level of anxiety at a given time.

Presence level and cybersickness were registered after each session in the VRT group using the Presence Questionnaire PQ v3.0 [33] and the Simulation Sickness Questionnaire (SSQ) [34]. The PQ consists of 32 items rated on a 7-point scale, assessing the participant’s perception of presence. The SSQ is a 16-item instrument on a 4-point scale that assesses the severity of motion sickness-related symptoms exhibited in a VE.

Heart rate (HR) was monitored and carried out throughout each session using a Polar (Kempele, Sweden) RS800CX™ device which has proved its efficacy in clinical research [35]. Heart rate variability (HRV) was assessed by calculating a time domain variable entitled root mean square of successive differences (RMSSD), which is the square root of the mean squared difference in successive RR or NN waves, and by the pNN50, which is the proportion of adjacent R or N waves that are more than 50 ms [36].

### 2.3. Apparatus and Virtual Environments

**Figure 1:**
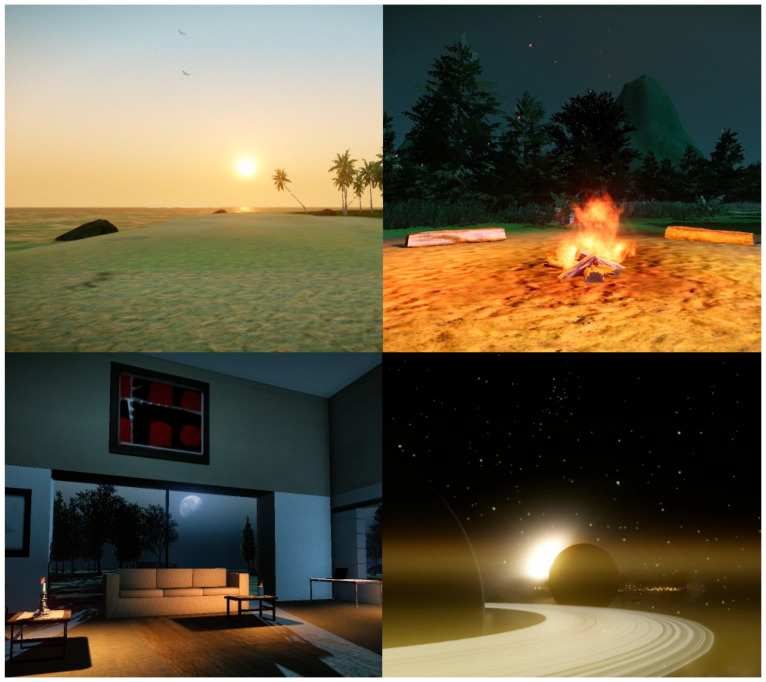
Screenshots of four VEs constructed for the present study. All VEs were interactive and the participant was free to walk, fly or swim anywhere and to stop at any place they considered fit to learn and practice relaxation.

The VR system included a Sensics^®^ (Columbia, NC, USA) zSight Head Mounted Display (HMD), coupled with a head motion tracker. The latter enabled the subject to visually explore the environment in a first-person view with the corresponding head movements performed in real time. A wireless remote control with a directional pad was also used, allowing various actions, from walking, to swimming to interacting with different 3D objects (such as opening doors). The software used to create and run the VEs was the commercially available game engine/level editor (GLE) CryEngine’s Sandbox (Crytek GmbH, Frankfurt, Germany). In order to propose diverse calming situations, the first author exploited the aforementioned GLE to build 6 specific VEs. At the beginning of each session, the VRT participants could select their chosen VE among these 6 relaxing virtual situations. These VEs were selected from guided imagery references [37] as well as pre-existing studies on the treatment of GAD in virtual reality [20]. The patients were offered the following virtual environments: a tropical island (deserted beach, calm sea with a few waves, sun setting on the horizon, waterfall); a forest (starry night, clearing with a campfire); polar ice fields (icebergs and smooth sea); an indoor environment (furnished apartment with candles and cushions and several large window bays); a journey across the solar system (departing near Earth, with possible interplanetary travel); and a sea of clouds (as seen from the top of a mountain, with clouds in motion). All VEs were elaborated as open worlds in order to convey a sense of freedom: participants were free to navigate (walk, fly or swim) at their leisure and stop at any place they considered fit to learn and practice relaxation (cf. Figure 1). Relaxing stereo sound effects specifically designed for each virtual environment (flowing water, slight breeze, chirping, etc.) could be heard. During the VR session, the participants also had the option to choose from 6 types of relaxation music belonging to the space music genre. Additionally, they had the possibility to select the time of day from four different ambiences (sunrise, noon, sunset, nighttime) as well as some meteorological phenomenon (wind, snowing). These parameters were implemented as they had been reported to have an impact on anxiety [38].

### 2.4. Procedures

**Figure 2:**
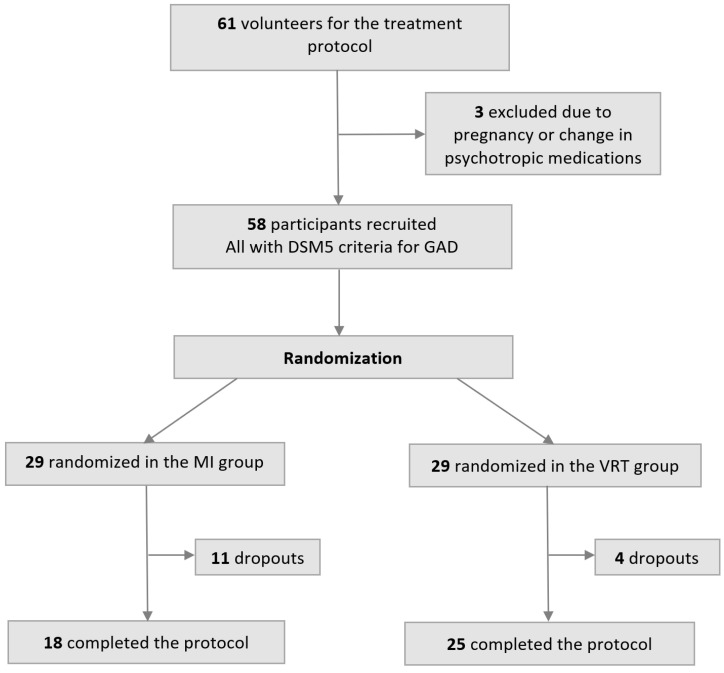
A flowchart of the different stages of inclusion and progression through the protocol.

Following the intake assessment and the diagnostic interview, the participants were randomly assigned to two therapeutic groups: VR relaxation therapy (VRT) and standard mental imagery relaxation therapy (MI). Participants were randomly assigned to each group using a computer-generated randomization list (cf. flowchart in Figure 2). The random sequence was generated via Random.org, which calculates true random numbers based on atmospheric noise, ensuring an unpredictable allocation. To enhance group comparability and control for potential confounders, randomization was stratified based on age and pre-treatment PSWQ and BDI scores. To ensure final comparability between pre-treatment samples, independent samples Student’s *t*-tests were performed between both groups. The analyses indicated no significant difference in scores for PSWQ: t(56) = 0.82, *p* = 0.42 (mean difference = 1.87, 95%, CI: −2.29 to 6.48), and for BDI t(56) = 0.61, *p* = 0.54 (mean difference = −0.95, 95%, CI: −4.10 to 2.19).

To minimize selection bias, allocation concealment was implemented by an independent researcher who was not involved in recruitment or assessment.

The protocol was composed of six 30 min sessions scheduled at a weekly rate for both groups. During the first three sessions and under a therapist’s supervision, all participants were taught relaxation techniques including respiratory exercises (session 1), Schultz’s autogenic training (session 2) [12] and Jacobson’s progressive muscular relaxation (session 3) [10]. From sessions 4 to 6, the participants were free to choose their practiced relaxation method.

For the MI group, participants were asked to imagine calming environments while learning or practicing their selected relaxation technique. In the VRT group, participants were invited to don an HMD and choose from 6 different VEs related to relaxation and detailed supra. Once selected, they decided on the time of day, meteorology and the presence or absence of music. They could then freely explore the VE until they found a place they considered agreeable and started learning or practicing a relaxation method.

Prior to immersion in MI or VR, baseline HR/HRV were measured at rest across 5 min in a seated position (baseline recording). To prevent baseline recordings from being contaminated by physiological disturbances due to potential anxiogenic thought processes common in GAD, the participants were asked to focus on the task of watching a neutral Japanese animation with subtitles. Self-report questionnaires were registered before and after the treatment procedure. SUDs and physiological measures were recorded during the MI or VRT sessions. The PQ and SSQ were completed after each VR relaxation session.

## 3. Results

A total of 15 participants dropped out at different stages of the protocol: 11 in the standard group due to difficulty in mentally visualizing relaxing places versus 4 in the VR group due to discomfort from donning the HMD for prolonged periods.

Regarding the choice of environments over the course of the 6 sessions in the VR group, 29.3% selected the tropical island, 18.7% the forest with a campfire, 16% the indoor environment, 14.7% the sea of clouds, 12.7% the space journey and 8.7% the North Pole. With respect to sound ambience and time of day, 68.9% of the participants chose music and 46.2% chose sunset, 24.5% night, 16.3% morning and 13% chose noon when this choice was possible. Lastly, in terms of relaxation method preference for the last three sessions and for both groups, 47.3% of the participants selected the Schultz autogenic training, 40.3% the progressive Jacobson’s muscular relaxation and 12.4% chose the vagal breathing.

### 3.1. Effects of Treatment on Symptoms and Physiology

The analysis followed an intention-to-treat approach. Dropout cases were included in the initial demographic characteristics but were excluded from the statistical calculations assessing treatment effects in both groups. Questionnaire scores, physiological measures and effect sizes are detailed in Table 1, Table 2 and Table 3. All pre- and post-treatment variables were assessed for normality using the Kolmogorov–Smirnov test, which indicated that the data were normally distributed (all *p*-values > 0.05), thereby meeting the assumptions required for subsequent parametric analyses.

**Figure 3:**
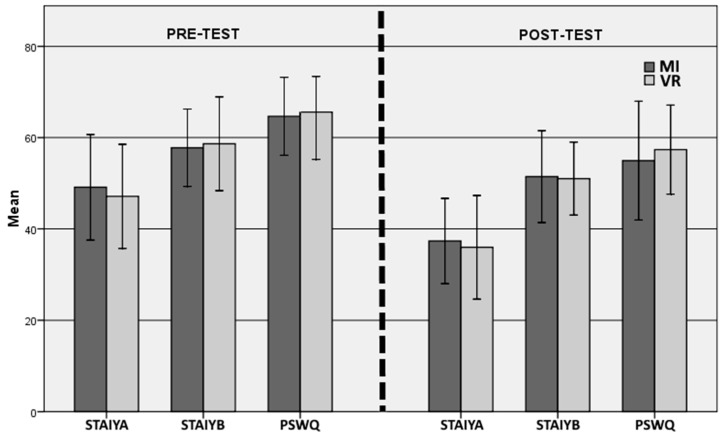
Means and standard deviations of dependent variables for pre-and post-test period (time). VR: virtual reality; MI: mental imaging; STAI-YA and -YB: State Trait Anxiety Inventory; PSWQ: Penn State Worry Questionnaire.

**Figure 4:**
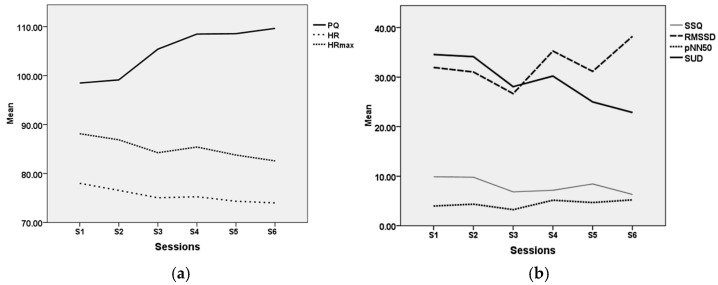
Line representation of mean PQ, SSQ, SUD, HR, RMSSD and pNN50 (**a**,**b**) across all sessions in VRT group (session 1 to 6). PQ: Presence Questionnaire; SSQ: Simulation Sickness Questionnaire; HR: mean heart rate per min (mean and maximum); RMSSD: root mean square of successive differences (ms); pNN50: proportion of adjacent R waves more than 50 ms (%), SUD: Subjective Unit of Discomfort.

A two-way mixed analysis of variance (ANOVA) was used to compare the two groups over time (pre vs. post). Significant main effects of time were demonstrated on the PSWQ, STAI-YA, STAI-YB, BDI-II, SF12 Mental and mean SUD scores, but not for SF12 Physical. Regarding group comparison, the examination of the group by time interactions indicated no significant interactions for any of these variables, indicating that the change in score related to the therapy was not different between the two groups. The mean scores, ANOVA and effect sizes are reported in Table 3 and Table 4 and illustrated in Figure 3. In addition, a significant decrease in anxiety as registered by the SUDs was observed over the course of the sessions in both groups (Table 2 and Table 3, and Figure 4b), but no difference was found between the groups.

Over the course of the six VRT sessions, the PQ scores tended to increase (Table 4 and Figure 4a) and the overall mean presence level was satisfactory (PQ = 104.94, SD = 20.59). Mean cybersickness was low (SSQ = 8.0053, SD = 5.54) (Table 4 and Figure 4b).

As for the analysis of the physiological recordings reported in Table 3, this was carried out on 27 participants only, due to several artifacts disrupting the HR data stream and hampering analysis. Concerning the two parameters of the HRV (RMSSD and pNN50), a significant increase was noted over time in the VR group, and there were no interactions between groups (Figure 4b). Although a decrease in the average HR over the sessions can be observed in both groups, it did not reach statistical significance.

### 3.2. Correlation

For systematic analysis, correlations were examined between demographic values. These results demonstrated a significant negative correlation between video game experience and age (r = −0.57), *p* < 0.00, and a significant positive correlation between treatment (psychotropic medication) and GAD duration (r = 0.39), *p* < 0.010.

Regarding presence in the VRT group, the influence of gaming experience on immersion was verified: there was minimal correlation between PQ and gaming experience (r = −0.05) *p* < 0.80. Moreover, to investigate any relationship between the perceived presence and the treatment efficacy, difference scores were calculated between the pre-test and the post-test values for all the dependent variables. Correlations were then calculated between these scores and the mean PQ scores across all sessions. Small to medium significant or non-significant positive correlations were observed between PQ and pre–post decrease for PSWQ (r = 0.17) *p* < 0.43, STAI-YB (r = 0.15) *p* < 0.47 and BDI (r = 0.43) *p* < 0.03. For STAI-YA and the quality of life ratings, only a minimal correlation was found between PQ and STAI-YB (r = 0.10) *p* < 0.61, SF12 mental (r = −0.12) *p* < 0.55 and SF 12 physical (r = 0.09) *p* < 0.67. With respect to the effect of presence on physiological parameters or point perceived anxiety, Pearson correlations were calculated between the PQ, SUDs and anxiety-related physiological measures recorded at all the VR sessions in the course of the treatment. Strong significant negative correlations were noted between PQ and mean SUDs (r = −0.89) *p* < 0.017, mean HR (r = −0.93) *p* < 0.007 and maximum HR (r = −0.90) *p* < 0.016. Strong significant positive correlations were found between PQ and RMSSD (r = 0.801) *p* < 0.05 and pNN50 (r = −0.84) *p* < 0.017. Lastly, and unsurprisingly, a large negative correlation was found between mean PQ and mean SSQ, which assesses the degree of cybersickness (r = −0.10) *p* < 0.60, a factor known to hamper presence [39].

## 4. Discussion

The present clinical trial shows that both interventions (VR and IM) are equivalently successful in mitigating anxiety and worry, which characterize GAD, as measured by the primary outcome and related psychometric scales. Notably, mood and the mental factor of mental quality of life also showed marked enhancement. In terms of questionnaire scores, while both groups evidenced significant reduction, group comparison did not lead to any significant interactions between any variables. This finding indicates that both VR and MI serve as suitable contexts for effectively implementing patients’ chosen relaxation techniques for GAD treatment, with neither method demonstrating superior therapeutic benefits in terms of perceived symptom reduction.

Recent findings [40] underscore the cerebellum’s pivotal role in constructing and updating internal models of sensorimotor relationships—not only for actions performed but also for those observed or imagined. This neurobiological mechanism may explain why both VR, which provides exposure to favorable environments, and MI, which relies on mental imagery of such contexts, are similarly effective in reshaping maladaptive sensory-motor patterns and reducing GAD symptoms. Future studies should further investigate this pathway to elucidate its contribution to therapeutic outcomes in anxiety disorders.

For the VR group, the study provides robust evidence that the relaxing and open-world VEs created with the GLE yielded clinical relaxation effects significantly detectable using physiological recordings, in addition to improvements observed in psychometric scores. Although average and maximum heart rate (HR) showed a downward trend across sessions, these changes did not reach statistical significance. In contrast, heart rate variability (HRV) parameters, specifically RMSSD and pNN50, demonstrated a significant increase over time in the VR group alone. Furthermore, the protocol completion rate was substantially higher in the VR group (86%) compared to the MI group (62%). Some participants in the MI group reported difficulties in mentally visualizing the relaxing scenes described by the therapist during sessions. This may have contributed to their lower completion rate. Conversely, participants in the VR group may have been more engaged and motivated, reflecting a broader trend observed in previous trials, where VR-based interventions for anxiety disorders consistently garnered higher user preference compared to traditional methods [41].

Concerning the potential influence of past exposure to digital entertainment, the correlations carried out on the demographic data revealed that the younger the participants, the more experience they had with video games; nonetheless, this experience had almost no effect on perceived presence in relaxing VEs. For involvement inside virtual environments, sought to ensure that participants behave in a VE as they would when exposed to similar calming cues in reality, the effect of presence is of significant importance [42]. In this study, the relaxing VEs yielded sufficient presence and calculations led to both significant and non-significant correlations between presence scores and self-reported questionnaire scores, as well as between presence scores and physiological parameters. These analyses point towards a possible relationship between the degree to which participants felt immersed inside the relaxing VEs and the therapeutic outcomes on depression, worry and a decrease in mean and maximum HR and increase in HRV. These findings emphasize the relevance of creating immersive multisensory experiences able to generate a high degree of presence with positive consequences on therapeutic efficacy. It is noteworthy to observe that undesirable side effects of VR, identified as cybersickness (nausea, sweating), may be the result of intervention by the parasympathetic system, a vegetative system known to produce HRV increase and similar symptoms [43]. However, the HR decrease and HRV increase over time while SSQ progressive reduction was occurring demonstrate that this evolution in the present physiological recordings is not linked to cybersickness.

With respect to the VRT participants’ modality preferences, it is of interest to mention that the virtual tropical beach and the forest with a campfire were the most frequently chosen environments for relaxation sessions. The preferred time of day in the VEs for these sessions was predominantly during sunset or nighttime, accompanied by space music. Since the right hemisphere of the brain plays a crucial role in both threat detection—implicated in GAD—and the processing of musical information [44,45], future studies could investigate the combined effects of music and VR on brain activity using neuroimaging techniques.

Finally, these participants tended to slightly favor the Schultz autogenic training over progressive Jacobson’s muscular relaxation during relaxation practice.

Overall, the potential therapeutic efficacy of VR for the treatment of GAD was demonstrated. While no significant differences were observed between the active VR group and the comparator (IM) in terms of clinical outcomes, VR had a notable positive effect on heart rate variability (HRV) and achieved a higher completion rate than IM in the recruited sample. It replicates similar findings from initial studies [20,42] and remains within the bounds of our intermediate analysis [46].

We will, nonetheless, exercise caution in our statements regarding the assessment of its clinical efficacy when compared to traditional methods and potential benefits given the technological and practical limitations of the present protocol. Firstly, the absence of olfactive stimuli (experimental scent banks can diffuse synchronous scents such as flowers, wet grass, ocean air, etc.) and thermal stimuli (obtained with specific immersive suits and useful when approaching the campfire for instance) might have restricted the multisensory integration of the relaxing cues present in the VEs. In a similar manner to real life, VEs stimulating several senses simultaneously may foster the multisensory integration of relaxing cues or related information [47]. Secondly, the clinical setting in itself may present a potential hurdle to achieving optimal results in VRT: wireless HMDs are becoming popular and can be used by the patients themselves in the familiar context of their home and under the optional supervision of a distant therapist as demonstrated by the effectiveness of home-based therapies such as neuromodulation [48]. Thirdly, recent advancements in miniaturization and hand motion capture have led to the development of more-comfortable and lightweight VR headsets, offering an enhanced VR experience tailored for relaxation in a resting position and without the use of controllers. Fourthly, the lack of blinding in this study may have introduced potential biases, as participants were aware of their assigned intervention as mentioned in the consent form, which could have influenced their subjective experiences and self-reported outcomes.

## 5. Conclusions

In fine, future studies centered on GAD should capitalize on the potential of lightweight, multisensory immersive equipment. In terms of therapeutic approaches, the superior outcomes on GAD symptoms achieved through integrated therapies combining cognitive behavioral therapy (CBT), relaxation and meditation/mindfulness with VR [26] highlight the relevance of combining other methods in addition to cycling and biofeedback. Furthermore, these findings point to the importance of exploring innovative opportunities afforded by artificial intelligence (AI), telepsychotherapy, telerelaxation in VR and group relaxation experiences within metaverses. Given that face-to-face interventions and instruction have the potential to amplify therapeutic effects in GAD [19], the development of AI-powered chatbots or conversational avatar agents based on fine-tuned open source large language models (LLMs) such as DeepSeek, Llama and Mistral and equipped with a virtual body and expressive facial features, represents a promising direction for advancing relaxation techniques. Moreover, ensuring ubiquitous access to VR environments, virtual relaxation places and to mental health professionals could prove to be critical in enabling individuals to effectively learn and apply relaxation strategies, irrespective of contextual, environmental, or spatial constraints. Such advancements could benefit individuals in a range of challenging scenarios, including confinement, remote or isolated locations, arctic research bases, deep-sea submarines, low-orbit space stations or even during prolonged voyages in outer space.

## Figures and Tables

**Table 1:** Social and demographic characteristics of sample. SD: standard deviation. MI: mental imagery; VRT: Virtual Reality Relaxation Therapy; anx.dis.: anxiety disorder; dep.: depression; OCD: Obsessive Compulsive Disorder; GAD: generalized anxiety.

Characteristics	Total *n* = 58 (SD)	VRT *n* = 29	MI *n* = 29
**Age** (years)	45.44 (12.75)	44.31 (12.89)	45.59 (12.82)
**Sex**			
Female	33	16	17
Male	25	13	12
**Marital Status**			
Single	20	9	11
Married/de facto	33	17	16
Divorced	4	3	1
Widower	1	0	1
**Professional status**			
Working	35	17	18
Unemployed	23	12	11
**Way of Life**			
Living alone	14	7	7
Living with a partner	20	8	12
Living with a family	21	12	9
Living inside a collectivity	3	2	1
**Education**			
Secondary	22	13	9
Tertiary	36	16	20
**Age of onset** (years)	37.69 (14.06)	36.03 (13.96)	39.34 (14.21)
**GAD duration** (years)	7.76 (8.09)	9.27 (9.25)	6.24 (6.56)
**Under psychotropic medication**			
Yes	26	14	12
No	32	15	17
**Comorbidity** (anx.dis., dep., OCD)			
0	23	14	9
1	19	6	13
2	16	9	7
**Video games experience**			
Playing every day	3	2	1
Playing more than once a week	5	2	3
Playing less than once a week	10	7	3
Stopped playing	18	8	10
Never played	22	10	12

**Table 2:** Means, standard deviations of dependent variables, results of two-way ANOVA between pre-and post-test period (time) and ANOVA for time × group comparison (interaction). ANOVA: analysis of variance; VR: virtual reality; MI: mental imaging; STAI-YA and -YB: State Trait Anxiety Inventory; PSWQ: Penn State Worry Questionnaire; BDI-II: Beck Depression Inventory version 2. SF-12 mental and physical quality of life questionnaire. *** *p* < 0.001; ** *p* < 0.025; ns: non-significant.

Tests	Gp	Pre-Test Mean (SD)	Post-Test Mean (SD)	ANOVA Time F(1,41)	Eta^2^ Time	ANOVA Inter. F(1,41)	Eta^2^ Inter.
**STAI-YA**	VR	47.12 (11.39)	35.96 (11.35)	10.26 **	0.30	0.18 ns	0.00
	IM	49.11 (11.56)	37.33 (9.33)	23.65 ***	0.58		
**STAI-YB**	VR	58.64 (10.28)	51.00 (7.99)	13.26 ***	0.36	0.163 ns	0.004
	IM	57.77 (8.48)	51.44 (10.06)	6.85 **	0.28		
**PSWQ**	VR	65.44 (8.46)	57.36 (9.75)	15.78 **	0.40	0.656 ns	0.16
	IM	64.66 (8.54)	54.94 (13.02)	12.80 **	0.43		
**BDI-II**	VR	10.76 (6.51)	7.16 (4.47)	8.78 **	0.27	1.027 ns	0.024
	IM	11.88 (6.00)	6.38 (5.15)	14.87 **	0.46		
**SF-12 Mental**	VR	31.63 (7.74)	37.76 (8.28)	8.38 **	0.26	2.297 ns	0.053
	IM	30.46 (7.99)	41.29 (10.27)	24.84 ***	0.59		
**SF-12 Phys.**	VR	47.83 (8.76)	46.67 (8.17)	0.67 ns	0.03	3.695 ns	0.083
	IM	45.46 (7.37)	48.43 (4.49)	3.50 ns	0.17		

**Table 3:** Means, standard deviations of dependent variables, results of two-way ANOVA between 1st session and 6th session (time) and ANOVA for time x group comparison (interaction). ANOVA: analysis of variance; VR: virtual reality; MI: mental imaging; SUD and HR S1 S6: mean Subjective Unit of Discomfort and heart rate per min during 1st session and 6th session; RMSSD: root mean square of successive differences (ms); pNN50: proportion of adjacent R waves more than 50 ms (%). ** *p* < 0.025; * *p* < 0.05; ns: non-significant.

Tests	Gp	S1 Mean (SD)	S6 Mean (SD)	ANOVA Time F(1,25)	Eta^2^ Time	ANOVA Inter. F(1,25)	Eta^2^ Inter.
**SUD**	VR	34.56 (18.89)	22.86 (16.40)	7.27 **	0.23	0.008 ns	0.00
**S1S6**	MI	35.74 (18.18)	24.63 (16.10)	5.57 *	0.24		
**HR**	VR	78.00 (19.24)	74.00 (15.49)	1.36 ns	0.09	0.094 ns	0.00
**S1S6**	MI	78.75 (18.13)	73.16 (15.43)	2.07 ns	0.16		
**RMSSD**	VR	21.28 (11.19)	31.70 (16.02)	10.07 **	0.42	1.90 ns	0.07
**S1S6**	MI	21.85 (10.57)	26.17 (10.92)	2.49 ns	0.18		
**pNN50**	VR	2.31 (2.82)	4.17 (4.08)	7.04 **	0.33	0.746 ns	0.03
**S1S6**	MI	2.48 (2.32)	3.05 (8.48)	1.86 ns	0.14		

**Table 4:** Means and standard deviations of subjective unit of discomfort (SUD), Presence Questionnaire (PQ) and Simulation Sickness Questionnaire (SSQ) for each therapeutic session.

Tests	Gp	S1 Mean (SD)	S2 Mean (SD)	S3 Mean (SD)	S4 Mean (SD)	S5 Mean (SD)	S6 Mean (SD)
**SUD**	VR	34.56 (18.89)	34.13 (13.24)	28.07 (14.83)	30.21 (19.04)	24.99 (15.91)	22.86 (16.40)
	MI	35.74 (19.18)	32.18 (15.84)	35.33 (13.08)	26.55 (20.74)	28.89 (20.18)	24.63 (16.10)
**PQ**	VR	98.48 (23.69)	99.12 (23.65)	105.40 (22.20)	108.48 (20.93)	108.56 (23.54)	109.64 (25.35)
**SSQ**	VR	9.81 (9.00)	9.80 (8.26)	6.84 (4.57)	7.16 (5.12)	8.44 (7.64)	6.32 (4.56)

## Data Availability

Data are available on demand from the corresponding author.

## Abbreviations

The following abbreviations are used in this manuscript:

CBT	Cognitive Behavioral Therapy
GAD	Generalized Anxiety Disorder
GLE	Game engine/Level Editor
HR	Heart Rate
HRV	Heart Rate Variability
MI	Mental Imagery
VR	Virtual Reality
VRET	Virtual Reality Exposure Therapy
VRT	Virtual Reality Relaxation Therapy

## References

[1] American Psychiatric Association (2013). Diagnostic and Statistical Manual of Mental Disorders.

[2] World Health Organization (2017). Depression and Other Common Mental Disorders: Global Health Estimates.

[3] Ruscio A.M., Hallion L.S., Lim C.C., Aguilar-Gaxiola S., Al-Hamzawi A., Alonso J., Scott K.M. (2017). Cross-sectional comparison of the epidemiology of DSM-5 generalized anxiety disorder across the globe. JAMA Psychiatry.

[4] World Health Organization (2010). International Statistical Classification of Diseases and Related Health Problems.

[5] Byers A.L., Yaffe K., Convinsky E., Friedman M.B., Bruce M.L. (2010). High occurrence of mood and anxiety disorders among older adults: The National Comorbidity Survey Replication. Arch. Gen. Psychiatry.

[6] Bereza B.G., Machado M., Einarson T.R. (2009). Systematic review and quality assessment of economic evaluations and quality-of-life studies related to generalized anxiety disorder. Clin. Ther..

[7] NICE (2011). Generalized Anxiety Disorder and Panic Disorder (With or Without Agoraphobia) in Adults: Management in Primary, Secondary and Community Care.

[8] Dugas M.J., Brillon P., Savard P., Turcotte J., Gaudet A., Ladouceur R. (2010). A randomized clinical trial of cognitive-behavioral therapy and applied relaxation for adults with generalized anxiety disorder. Behav. Ther..

[9] Clark D., Salkovkis P., Chalkley A. (1985). Respiratory control as a tretment for panics attacks. J. Behav. Ther. Exp. Psychiatry.

[10] Jacobson E. (1938). Progressive Relaxation.

[11] Öst L.G. (1988). Applied Relaxation: Description of an Effective Coping Technique. J. Behav. Ther. Exp. Ther..

[12] Schultz J.H., Luthe W. (1969). Autogenic Training.

[13] Hoge E.A., Bui E., Marques L., Metcalf C.A., Morris L.K., Robinaugh D.J., Simon N.M. (2013). Randomized controlled trial of mindfulness meditation for generalized anxiety disorder: Effects on anxiety and stress reactivity. J. Clin. Psychiatry.

[14] Manzoni G.M., Pagnini F., Castelnuovo G., Molinari E. (2008). Relaxation training for anxiety: A ten-years systematic review with meta-analysis. BMC Psychiatry.

[15] Taylor J.H., Lebowitz E.R., Jakubovski E., Coughlin C.G., Silverman W.K., Bloch M.H. (2018). Monotherapy Insufficient in Severe Anxiety? Predictors and Moderators in the Child/Adolescent Anxiety Multimodal Study. J. Clin. Child Adolesc. Psychol..

[16] Schröder D., Wrona K.J., Müller F., Heinemann S., Fischer F., Dockweiler C. (2023). Impact of virtual reality applications in the treatment of anxiety disorders: A systematic review and meta-analysis of randomized-controlled trials. J. Behav. Ther. Exp. Psychiatry.

[17] Meyerbröker K., Morina N. (2021). The use of virtual reality in assessment and treatment of anxiety and related disorders. Clin. Psychol. Psychother..

[18] Alahmari K., Duh H., Skarbez R. (2023). Outcomes of virtual reality technology in the management of generalised anxiety disorder: A systematic review and meta-analysis. Behav. Inf. Technol..

[19] Fodor L.A., Coteț C.D., Cuijpers P., Szamoskozi Ș., David D., Cristea I.A. (2018). The effectiveness of virtual reality based interventions for symptoms of anxiety and depression: A meta-analysis. Sci. Rep..

[20] Gorini A., Riva G. (2008). The potential of virtual reality as anxiety management tool: A randomized controlled study in a sample of patients affected by generalized anxiety disorder. Trials.

[21] Gorini A., Pallavicini F., Algeri D., Gaggioli A., Riva G. (2010). Virtual reality in the treatment of generalized anxiety disorders. Stud. Health Technol. Inform..

[22] Guitard T., Bouchard S., Bélanger C., Berthiaume M. (2019). Exposure to a Standardized Catastrophic Scenario in Virtual Reality or a Personalized Scenario in Imagination for Generalized Anxiety Disorder. J. Clin. Med..

[23] Keshavarz N., Abad T.N., Beyrami M., Alilou M.M., Roudsari A.B. (2021). Efficacy of Virtual Reality Based Worry Exposure Therapy on the Anxiety Severity and Worry in Generalized Anxiety Disorder. Adv. Biosci. Clin. Med..

[24] Grassi A., Gaggioli A., Riva G. (2009). The green valley: The use of mobile narratives for reducing stress in commuters. Cyberpsychol. Behav. Soc. Netw..

[25] Wang T.C., Tsai C.L., Tang T.W., Wang W.L., Lee K.T. (2019). The Effect of Cycling Through a Projection-Based Virtual Environment System on Generalized Anxiety Disorder. J. Clin. Med..

[26] Popa C.O., Sava F.A., Muresan S., Schenk A., Cojocaru C.M., Muntean L.M., Olah P. (2022). Standard CBT versus integrative and multimodal CBT assisted by virtual-reality for generalized anxiety disorder. Front. Psychol..

[27] Lombard M., Ditton T. (1997). At the heart of it all: The concept of presence. J. Comput.-Mediat. Commun..

[28] Spielberger C.D. (1983). Manual for the STAI.

[29] Beck A.T., Steer R.A., Brown T.A. (1996). Beck Depression Inventory Manual.

[30] Meyer T.J., Miller M.L., Metzger R.L., Borkovec T.D. (1990). Development and validation of the Penn State Worry Questionnaire. Behav. Res. Ther..

[31] Gandek B., Ware J.E., Aaronson N.K. (1998). Crossvalidation of item selection and scoring for the SF-12 Health Survey in nine countries: Results from the IQOLA Project International Quality of Life Assessment. J. Clin. Epidemiol..

[32] Wolpe J. (1969). The Practice of Behavior Therapy.

[33] Witmer B.G., Singer M.J. (1998). Measuring Presence in Virtual Environments: A presence Questionnaire. Presence Teleoper. Virtual Environ..

[34] Kennedy R.S., Lane N.E., Berbaum K.S., Lilienthal M.G. (1993). Simulator Sickness Questionnaire: An Enhanced Method for Quantifying Simulator Sickness. Int. J. Aviat. Psychol..

[35] Quintana D.S., Heathers J.A., Kemp A.H. (2012). On the validity of using the Polar RS800 heart rate monitor for heart rate variability research. Eur. J. Appl. Physiol..

[36] Vanderlei L.C.M., Silva R.A., Pastre C.M., Azevedo F.M., Godoy M.F. (2008). Comparison of the Polar S810i Monitor and the ECG for the Analysis of Heart Rate Variability in the Time and Frequency Domains. Braz. J. Med. Biol. Res..

[37] Van Kuiken D. (2004). A meta-analysis of the effect of guided imagery practice on outcomes. J. Holist. Nurs..

[38] Marks I. (1987). Fears, Phobias and Rituals.

[39] Weech S., Kenny S., Barnett-Cowan M. (2019). Presence and Cybersickness in Virtual Reality Are Negatively Related: A Review. Front. Psychol..

[40] Antonioni A., Raho E.M., Straudi S., Granieri E., Koch G., Fadiga L. (2024). The cerebellum and the Mirror Neuron System: A matter of inhibition? From neurophysiological evidence to neuromodulatory implications. A narrative review. Neurosci. Biobehav. Rev..

[41] Garcia-Palacios A., Botella C., Hoffman H. (2007). Comparing acceptance and refusal rates of virtual reality exposure Vs. in vivo exposure by patients with specific phobias. Cyberpsychol. Behav..

[42] Villani D., Riva G. (2008). Presence and relaxation: A preliminary controlled study. PsychNol. J..

[43] Goldberger J., Challapalli S., Tung R., Parker M.A., Kadish A.H. (2001). Relationship of Heart Rate Variability to Parasympathetic Effect. Circulation.

[44] Hartikainen K.M. (2021). Emotion-Attention Interaction in the Right Hemisphere. Brain Sci..

[45] Harms V., Cochran C., Elias L. (2014). Melody and Language: An Examination of the Relationship Between Complementary Processes. Open Psychol. J..

[46] Malbos E., Chichery N., Borwell B., Seimandi J., Weindel G., Lançon C. (2020). Virtual reality for relaxation in the treatment of generalized anxiety disorder: A comparative trial. ARCTT Annu. Rev. Cyberther. Telemed..

[47] Stein B.E., Meredith M.A. (1993). The Merging of the Senses.

[48] Woodham R.D., Selvaraj S., Lajmi N., Hobday H., Sheehan G., Ghazi-Noori A.-R., Lagerberg P.J., Rizvi M., Kwon S.S., Orhii P. (2025). Home-based transcranial direct current stimulation treatment for major depressive disorder: A fully remote phase 2 randomized sham-controlled trial. Nat. Med..

